# Health-related quality of life as predictor of mortality in end-stage renal disease patients: an observational study

**DOI:** 10.1186/s12882-019-1318-x

**Published:** 2019-04-29

**Authors:** Ming Pei, Rute Aguiar, Agneta A. Pagels, Olof Heimbürger, Peter Stenvinkel, Peter Bárány, Charlotte Medin, Stefan H. Jacobson, Britta Hylander, Bengt Lindholm, Abdul Rashid Qureshi

**Affiliations:** 1 Renal Medicine and Baxter Novum, Clintec, M99, Karolinska University Hospital Huddinge, 141 86 Stockholm, Sweden; 2 First Teaching Hospital of Tianjin University of Traditional Chinese Medicine, Tianjin, China; 3 Department of Nephrology, Hospital Espírito Santo, Évora, Portugal; 4 Department of Nephrology, Karolinska Institutet and Karolinska University Hospital Solna, Stockholm, Sweden; 5 Department of Clinical Sciences, Division of Nephrology, Danderyd University Hospital, Karolinska Institutet, Stockholm, Sweden

**Keywords:** Health-related quality of life, Chronic kidney disease, Patient-centered outcomes, Peritoneal dialysis, Hemodialysis, Mortality

## Abstract

**Background:**

Health-related quality of life (HRQoL) is an important component of patient-centered outcomes and a useful parameter for monitoring quality of care. We assessed HRQoL, its determinants, and associations with mortality in patients with end-stage renal disease (ESRD).

**Methods:**

Short Form-36 was used to assess HRQoL, its domain components, and physical (PCS) and mental (MCS) composite summary scores in altogether 400 (338 incident and 62 prevalent) dialysis patients with median age 64 years, 37% women, 24% diabetes mellitus (DM), 49% cardiovascular disease (CVD), and median estimated glomerular filtration rate (eGFR) of 5.3 (3.0–9.4) ml/min/1.73^2^. Results were analyzed separately for 338 incident patients starting on hemodialysis (HD; 68%) or peritoneal dialysis (PD; 32%), and 62 prevalent PD patients. Mortality risk was analyzed during up to 60 months (median 28 months).

**Results:**

Linear multivariate regression analysis showed that in incident dialysis patients, 1-SD higher PCS associated negatively with 1-SD higher age, DM and CVD, and positively with 1-SD higher hemoglobin and sodium (adjusted *r*^2^ = 0.17). In 62 prevalent PD patients, 1-SD higher PCS was negatively associated with 1-SD higher age. MCS was not associated to any of the investigated factors. Multivariate Cox regression analysis showed that in incident dialysis patients, 1-SD increase of PCS associated with lower all-cause mortality, hazard ratio 0.65 (95% confidence interval 0.52–0.81), after adjustments for age, sex, DM, CVD, plasma albumin, C-reactive protein and eGFR whereas 1-SD lower MCS did not associate with mortality. In PD patients, neither PCS nor MCS associated with mortality.

**Conclusions:**

MCS did not associate with any of the investigated clinical factors, whereas lower PCS associated with higher age, CVD, DM, and lower hemoglobin and sodium levels. MCS was not associated with mortality, whereas lower PCS associated with increased mortality risk. These results suggest that HRQoL - in addition to its role as patient-centered outcome - matters also for hard clinical outcomes in ESRD patients. Our knowledge about factors influencing MCS in ESRD patients is limited and should motivate further studies.

**Electronic supplementary material:**

The online version of this article (10.1186/s12882-019-1318-x) contains supplementary material, which is available to authorized users.

## Background

Chronic kidney disease (CKD) associates with significant clinical, societal and psychosocial burdens, especially in end-stage renal disease (ESRD) patients, and - due to cardiovascular disease, infections, protein-energy wasting and premature aging [1, 2] - to high morbimortality [3]. Health-related quality of life (HRQoL) is an important aspect of patient-centered clinical outcomes [4, 5], and assessment of HRQoL is increasingly used as outcome measure when monitoring the quality and effectiveness of renal care including maintenance dialysis treatment [6–8].

HRQoL usually deteriorates during the course of CKD progression [9, 10] and is often markedly reduced in ESRD patients due to restrictions affecting life style and food intake, multiple medications, effects of dialysis therapy, disease-related complications [11], common comorbidities [12, 13], the uremic milieu [14, 15] and premature aging [1, 2]*.* Efforts aiming at improving HRQoL are warranted not only because satisfactory HRQoL is a goal in itself but also because poor HRQoL is linked to hospitalization and mortality [6, 16–19].

Many factors influencing HRQoL in ESRD patients have been identified [10, 20, 21] such as country, ethnicity, and demographic variables [22]. HRQoL was investigated in ESRD patients on hemodialysis (HD) or peritoneal dialysis (PD) in Korea [23], Singapore [24], Brazil [18, 25], US [26], Turkey [27], Netherlands [28, 29], South Africa [30] and Sweden [10]. The impact of dialysis modality on HRQoL has been analyzed [31]. However, few larger studies evaluated the mortality predictive role of HRQoL in ESRD patients.

In the present study, we investigated factors associated with HRQoL and analyzed associations of HRQoL and its components with survival in 338 Swedish ESRD patients starting dialysis on HD (≈68%) or PD (≈32%), and in 62 prevalent PD patients. The two cohorts are analysed and presented separately.

## Methods

### Patients

This study is a post hoc analysis of available Short Form-36 (SF-36) scores and clinical data for two separate cohorts comprising incident (*n* = 338) and prevalent (*n* = 62) dialysis patients. SF-36 assessments in 8930 individuals (aged 15–93 years; 48% males) representing the Swedish population [32, 33] served as reference for comparative analyses.

#### Incident dialysis patients

CKD stage 5 patients (*n* = 338; median age 63 years; 31% women) initiating dialysis with HD (≈68%) or PD (≈32%) were recruited during 2000–2005 within a longitudinal study (PAUS, Prospective study of renal replacement therapy in Stockholm) at the Karolinska University Hospital and Danderyd Hospital, Stockholm, Sweden. The protocol and part of the data from the PAUS study have been described earlier [10]. The current study includes those patients with measurements of HRQoL. Each patient’s medical chart was thoroughly reviewed by a nephrologist, and data was extracted on underlying kidney disease, presence of clinically overt cardiovascular disease (CVD), and other comorbid conditions such as diabetes mellitus (DM). The causes of CKD were diabetic nephropathy (25%), hypertension/renal vascular disease (20%), chronic glomerulonephritis (13%), and other (such as polycystic kidney disease), or unknown, etiologies (42%). Mortality risk was analyzed during up to 60 months, median 29.6 months.

#### Prevalent dialysis patients

Prevalent PD patients (*n* = 62; median age 65 years; 35% women) were recruited during 2008–2011 within a study (MIMICK2, Mapping of Inflammation Markers in Chronic Kidney Disease 2) aiming at including all PD patients controlled at the Karolinska University Hospital and Danderyd Hospital, Stockholm, Sweden. The protocol and part of the data from the MIMICK2 study have been described earlier [34]. Briefly, 84 out of 163 invited patients agreed to participate; however, HRQoL data were lacking in 22 patients. The remaining 62 patients had undergone PD for median 12.1 (10-90th percentile range 4.1–40.0) months. Mortality risk was analyzed during up to 60 months, median 22.2 months.

The Ethics Committee of Karolinska Institute at Karolinska University Hospital, Stockholm, approved the study protocols and each patient gave their written informed consent.

### SF-36 questionnaire

The Medical Outcomes Study Short Form-36 (SF-36) is a self-report generic instrument for assessment of two major aspects of patients’ HRQoL and includes 36 items that measures eight dimensions, or health domains, on a 100-point scale [35]. The eight domains are: 1. Physical functioning (PF), 2. Role limitations caused by physical problems (RP), 3. Bodily pain (BP), 4. General health (GH), 5. Vitality/energy/fatigue (VT), 6. Mental health/emotional well-being (MH), 7. Role limitations caused by mental health/emotional problems, and 8. Social functioning (SF). Results from the questionnaire were summarized into a *physical composite summary (PCS)* score aggregating items from PF, RP, BP, and GH, and a *mental composite summary (MCS)* score aggregating items from the domains VT, SF, MH, and RE. In the Swedish normative reference population, mean values of these scores were: PF = 87.9, RP = 83.2, BP = 74.8, GH = 75.8, VT = 68.8, SF = 88.6, MH = 80.9, RE = 85.7, and PCS = 50 and MCS = 50 [33, 36].

HRQoL was assessed at dialysis initiation, or up to 2 weeks after the first dialysis session among incident dialysis patients, and at baseline in the prevalent dialysis patients.

### Clinical assessment

Height and body weight were obtained at baseline and body mass index (BMI) was recorded. Arterial systolic and diastolic blood pressures (BP) were measured in the morning after a 15-min resting period, and mean BP (1/3 * systolic BP) + (2/3*diastolic BP) [37] were recorded. Comorbidity was assessed using Davies comorbidity score [38].

### Biochemical analyses

Blood samples, collected in incident dialysis patients at baseline before commencement of regular maintenance dialysis, and in PD patients after an overnight oral fast without interrupting ongoing PD, were separated to obtain plasma within 30 min, and samples were kept frozen at − 70 °C if not analyzed immediately. Concentrations of hemoglobin and serum albumin (bromcresol purple), creatinine, potassium, calcium, phosphate, parathyroid hormone (PTH), C-reactive protein (CRP) were determined by routine methods at the Department of Laboratory Medicine, Karolinska University Hospital. Plasma interleukin-6 (IL-6) analyzed by commercial kits on Immulite Automatic Analyzer (Siemens Medical Solutions, Los Angeles, CA, USA) according to the instructions of the manufacturer. Data on glomerular filtration rate (GFR) were not available in all patients. Instead estimated GFR (eGFR) was calculated based on the CKD-EPI equation [39], also in PD patients as described previously [40].

### Statistical analysis

Data are expressed as median and range (10th – 90th percentile) or percentage, as appropriate. Statistical significance was set at the level of *p* < 0.05. Comparisons between two groups were assessed with the nonparametric Wilcoxon test for continuous variables and Fischer’s exact test or Chi-square test for nominal variables. Spearman’s rank correlation analysis was used to determine associations between selected parameters. The differences in HRQoL domains and summary scores between the combined cohort of 400 ESRD patients and the Swedish reference population (*n* = 8930) were assessed using Cohen’s effect size d, computed according to Becker [32]. Cohen’s effect size (d), a standardized measure of effect size (ES), was defined as the differences between the mean values of two samples divided by the pooled within-group standard deviation (SD). An effect size of one is equivalent to change of one SD between the groups. According to Cohen, benchmarks for interpreting the magnitude of differences are: ES values< 0.49 are considered as small, values of 0.50–0.79 as medium, and values ≥0.80 are considered as large. Principal component analysis was used to analyze the construct validity for the two summary scales (PCS and MCS), i.e., the degree by which these scales measured what these scales were purported to be measuring when using SF-36. Cox proportional hazard models were used to obtain hazard ratios (HRs) for all-cause mortality risk for PCS and MCS, adjusting for all confounders. As there were relatively few missing values (see Table 1), imputations were not used. Statistical analyses were performed using statistical software SAS version 9.4 (SAS Campus Drive, Cary, NC, USA) and Stata 15.1 (Stata Corporation, College Station, TX, USA).

**Table 1:** Baseline demographic and biochemical characteristics of the separate cohorts of incident dialysis patients and prevalent PD patients, respectively

Baseline characteristics		Incident dialysis^a^ (*n* = 338)	Prevalent PD (*n* = 62)	*p*
Age (years; *n* = 338/62)		63 (45–80)	65 (49–81)	NS
Female (%; *n* = 338/62)		105 (31)	23 (35)	NS
CVD (%; *n* = 336/62)		177(53)	19 (31)	**0.001**
DM (%; *n* = 338/62)		84 (25)	13 (21)	NS
Davies Comorbidity Score (%; *n* = 335/62)	Low risk (0)	124 (37)	19 (31)	NS
	Medium risk (1–2)	150 (45)	34 (55)	
	High risk (≥3)	61 (18)	9 (15)	
BMI (kg/m^2^; *n* = 338/62)		25 (22–28)	25 (23–28)	NS
MBP (mmHg, *n* = 333/62)		103 (85–126)	97.0 (80–117)	**0.0004**
eGFR^b^(ml/min, *n* = 338/62)		4.9 (2.9–9.6)	5.8 (4.5–9.2)	**0.0004**
Weekly KT/V (renal + peritoneal)		NA	2.2(1.7–2.9)	–
Metabolic biomarkers				
Hemoglobin (g/L, *n* = 338/62)		109 (89–127)	121 (106–132)	**< 0.0001**
Albumin (g/L; *n* = 336/62)		32 (25–39)	32 (24.6–38)	NS
Potassium (mmol/L, *n* = 338/62)		4.4 (3.5–5.4)	4.1 (3.2–4.9)	**0.0002**
Ca x P (*n* = 337/62)		4.5 (2.8–6.5)	3.9 (2.6–5.5)	**< 0.0001**
PTH (ng/L, *n* = 327/62)		200 (45–595)	256 (78–567)	**0.03**
CRP (mg/L; *n* = 333/62)		8(5.1–75.6)	4.1(0.7–26.2)	**0.002**
IL-6 (pg/mL; *n* = 147/62)		5.6 (2.1–29.0)^c^	6.4 (2.1–21.7)	NS

The *p* values are noted for descriptive purpose only

Presented as median and range (10th – 90th percentile), numbers, or proportions

Bold entries are significant

*Abbreviations*: *CVD* cardiovascular disease, *DM* diabetes mellitus, *BMI* body mass index, *MBP* mean blood pressure, *eGFR* estimated glomerular filtration rate, *Ca* calcium, *P* phosphate, *CRP* C-reactive protein, *IL-6* interleukin-6, *PTH* parathyroid hormone

^a^Incident dialysis patients included patients starting on HD (≈68%) and PD (≈32%)

^b^eGFR, according to CKD-EPI equation

^c^Variable measured in 147 out of 338 Incident dialysis patients

## Results

Baseline characteristics of the 338 incident dialysis patients and 62 prevalent PD patients are summarized in Table 1. While age, sex distribution and Davies comorbidity score did not differ, PD patients had lower CVD prevalence, lower mean blood pressure, higher eGFR, higher hemoglobin, lower potassium, lower calcium x phosphate product and higher PTH levels than the incident dialysis patients.

### HRQoL as assessed by SF-36

HRQoL data in the 338 incident dialysis patients and 62 prevalent PD patients are presented in Table 2. PD patients scored better in all domains, except General Health, and had higher MCS score and PCS tended to be higher (*p* = 0.06) compared with the incident dialysis patients.

**Table 2:** The eight domains of Short Form-36 (SF-36) measures of HRQOL, and results summarized as PCS and MCS in the 2 cohorts, 338 incident dialysis patients and 62 prevalent PD patients

HRQOL	Domains	Incident dialysis (*n* = 338)	Prevalent PD (*n* = 62)	*p*
PF	Physical Functioning	50 (5–85)	60 (20–93.5)	**0.0129**
RP	Role Physical	0 (0–75)	25 (0–100)	**0.0001**
BP	Bodily Pain	60.5 (12–100)	73 (31.3–100)	**0.0193**
GH	General Health	40 (20–65.2)	40 (21.5–69.1)	0.2748
VT	Vitality	32.5 (5–70)	50 (15–75)	**< 0.0001**
SF	Social Functioning	50 (12.5–100)	75 (28.8–100)	**0.0003**
RE	Role Emotional	0 (0–100)	66.7 (0–100)	**0.0043**
MH	Mental Health	64.0 (28–92)	84 (50.4–96)	**< 0.0001**
MCS	Mental composite summary	35.7 (21.7–56.5)	46.9 (27.0–58.8)	**< 0.001**
PCS	Physical composite summary	31.8 (19.1–47.1)	33.0 (23.0–49.1)	0.0547

Presented as median and (10th – 90th percentile)

Bold entries are significant

### Univariate correlations between PCS, MCS and clinical characteristics and investigated biomarkers

In 338 incident dialysis patients, PCS and MCS correlated with serum albumin (rho = 0.17, *p* < 0.001; rho = 0.12, *p* < 0.05) and hemoglobin (rho = 0.19, *p* < 0.001; rho = 0.20, *p* < 0.00), respectively, and negatively with CRP (PCS: rho = − 0.22, *p* < 0.001; MCS: rho = − 0.15, *p* < 0.01, respectively). Furthermore, PCS associated with comorbidities (CVD and DM), BMI (rho = − 0.11, *p* < 0.05), and sodium (rho = 0.15, *p* < 0.01), while MCS correlated with serum calcium (rho = 0.14, *p* < 0.05) and male gender (rho = − 0.13, *p* < 0.01). There were only a few statistically significant associations in the PD cohort.

### Differences between ESRD patients and normative population expressed as effect sizes

The results for calculated effect sizes (ES) of differences between in 338 incident dialysis patients, 62 prevalent PD patients and the normative Swedish population are presented in Table 3 [33, 36]. Briefly, most dimensions and PCS (ES = -0.67) differed as compared with the normative subjects in the incident dialysis patients with differences being of medium magnitude (− 0.50 to − 0.79). However, Bodily pain and Mental health, as well as MCS (ES = -0.46) showed differences of small magnitude (ES − 0.50 to − 0.79).

**Table 3:** Effect sizes (ES) in HRQoL domains and summary scores in prevalent PD patients and incident dialysis patients compared with the normative Swedish population (*n* = 8930)

SF-36	Incident dialysis		Prevalent PD	
	Cohen’s d	Effect-size r	Cohen’s d	Effect-size r
PF	−1.67	−0.64	−1.36	− 0.64
RP	−2.00	−0.71	− 1.39	− 0.71
BP	− 0.57	− 0.27	− 0.23	− 0.27
GH	− 1.72	− 0.65	− 1.55	− 0.65
VT	− 1.50	− 0.60	− 0.89	− 0.60
SF	−1.33	− 0.55	− 0.90	− 0.55
RE	− 1.35	− 0.56	− 0.88	− 0.56
MH	− 0.89	− 0.41	− 0.22	− 0.41
PCS^a^	−1.80	−0.67	− 1.47	− 0.67
MCS^a^	− 1.04	− 0.46	− 0.44	−0.46

Interpretation of effect size (ES): ES of small magnitude < 0.49, medium sized ES > 0.50 and large sized ES > 0.80

*Abbreviations*: *SF-36* short form-36, *PF* Physical functioning, *RP* Role physical, *BP* Bodily pain, *GH* General health, *VT* Vitality, *SF* Social functioning, *RE* Role emotional, *MH* Mental health, *PCS* Physical composite summary score, *MCS* Mental composite summary score

^a^For comparisons with summary scores PCS and MCS of normative Swedish population, *n* = 8004

In contrast, prevalent PD patients demonstrated, compared to the normative Swedish population, smaller differences and medium magnitude for differences (ES − 0.50 to − 0.79) were observed only for three dimensions, Physical functioning, Role physical, and General health as well as for PCS (ES = -0.59) whereas for the other domains, and for MCS (ES = -0.21), differences were of small ES magnitude.

No dimensions reached large-sized ES difference (≥0.80) in any of the above-mentioned analyses.

### Multivariate analysis of parameters associated with PCS and MCS

Linear multivariate regression analysis showed that among the 338 incident dialysis patients, 1-SD higher PCS associated negatively with 1-SD higher age, DM and CVD, and positively with 1-SD higher Hb and Na (adjusted *r*^2^ = 0.17). In 62 prevalent PD patients, 1-SD higher PCS was negatively associated with 1-SD higher age, see Table 4.

**Table 4:** Linear multiple regression models for 1-SD of PCS and 1-SD of MCS in incident dialysis and prevalent dialysis patients

	Incident dialysis		Prevalent PD	
	1-SD of PCS	1-SD of MCS	1-SD of PCS	1-SD of MCS
	(β, *P*) (*r*^2^ = 0.17)	(β, *P*) (*r*^2^ = 0.04)	(β, *P*) (*r*^2^ = 0.17)	(β, *P*) (*r*^2^ = 0.05)
1-SD of age, years	**−0.11 (0.04)**	0.11 (0.07)	**−0.40 (0.002)**	0.03 (0.81)
Gender female vs male	0.05 (0.32)	−0.05 (0.31)	−0.03 (0.82)	− 0.02 (0.88)
DM absence vs presence	**−0.15(0.006**)	− 0.10(0.08)	−0.12(0.33)	− 0.001(0.99)
CVD absence vs presence	**−0.25(0.001)**	− 0.06(0.32)	−0.02 (0.85)	− 0.04(0.78)
1-SD of albumin, g/L	0.04(0.49)	0.07(0.19)	0.11(0.38)	0.04(0.79)
1-SD of hsCRP, mg/L	−0.01(0.89)	−0.03(0.52)	− 0.17(0.15)	0.11(0.45)
1-SD of Hb, g/L	**0.13(0.01)**	0.11(0.06)	0.07(0.55)	0.12(0.41)
1-SD of Na, mmol/L	**0.14(0.01)**	0.06(0.24)	0.04(0.72)	0.19(0.20)

Statistically significant associations are marked as bold

*Abbreviations*: *SD* standard deviation, *DM* diabetes mellitus, *CVD* cardiovascular disease, *hsCRP* high sensitivity C-reactive protein, *Hb* hemoglobin

Linear multivariate regression analysis showed that there were no significant associations of 1-SD higher MCS with clinical variables among the 338 incident dialysis patients and 62 prevalent PD patients; see Table 4.

### Multivariate competing risk analysis for all-cause mortality

In 338 incident dialysis patients during follow-up, median 29 months, 116 (34%) out of 338 patients died and 83 (24%) underwent renal transplantation. In 62 prevalent dialysis patients during follow-up, median 22 months, 28 (45%) out of 62 patients died and 14 (23%) underwent renal transplantation. Multivariate Cox regression analysis for all-cause mortality, taking renal transplantation into account, and with patients with ≥ median of PCS and MCS serving as reference group, showed statistically significant difference for PCS only in incident dialysis patients. Briefly, in incident dialysis patients, 1-SD lower PCS associated with increased mortality whereas 1-SD lower MCS did not associate with mortality. In PD patients, neither PCS nor MCS associated with mortality (Tables 5 and 6).

**Table 5:** Cox proportional hazard model for all-cause mortality of PCS (< 31.8 versus > = 31.8) in 338 incident dialysis patients (A) and PCS (< 33.0 versus > = 33.0) in prevalent dialysis patients (B). Data are shown as 1-SD increase of PCS. Data are expressed as hazard ratios (HR) and 95% confidence intervals (95%CI)

	(A) Incident dialysis patients		(B) Prevalent PD patients	
	HR	*p* value	HR	*p* value
PCS < median vs > = median in each cohort	**0.62 (0.42–0.93)**	**0.02**	0.42 (0.13–2.54)	0.47
1-SD increase of PCS	**0.65 (0.52–0.81)**	**< 0.001**	0.75 (0.32–1.75)	0.51

Bold entries are significant

**Table 6:** Cox proportional hazard model for all-cause mortality of MCS (< 35.7 versus > = 35.7) in 338 incident dialysis patients (A) and MCS (< 46.9 versus > = 46.9) in prevalent dialysis patients (B). Data are shown as 1-SD increase of MCS. Data are expressed as hazard ratios (HR) and 95% confidence intervals (95%CI)

	(A) Incident dialysis patients		(B) Prevalent PD patients	
	HR	*p* value	HR	*p* value
MCS < median vs > = median in each cohort	0.80 (0.54–1.18)	0.26	1.41 (0.40–4.91)	0.59
1-SD increase of MCS	0.96 (0.79–1.17)	0.71	1.31 (0.55–3.12)	0.52

Adjustments for confounders included age, gender, presence of diabetes and cardiovascular disease, levels of plasma albumin, high-sensitivity C-reactive protein and eGFR

*Abbreviations*: *SD* standard deviation, *PCS* physical composite summary score, *MCS* mental composite summary score

### Principal component analysis

Principal component analysis was performed to ascertain the construct validity of the SF-36 results. Briefly, in 338 incident dialysis patients the physical components (PCS) was found to explain 75.2% whereas mental components (MCS) explained 74.2% whereas in 62 prevalent PD patients, PCS explained 35.2% and MCS 43.4% of the variance in HRQoL measurements (Additional file 1: Table S1).

## Discussion

It is easy to lose sight of the fact that for patients their mental health and satisfaction with their treatment are as important—if not more important—than meeting clinical or numerical laboratory targets [12]. Here we report that in ESRD patients, lower HRQoL - a key measure of patient-centered outcomes [5] - was associated with increased all-cause mortality, indicating that quality of life matters also for hard outcomes of ESRD patients. To the best of our knowledge, the present study is - together with [10] - the largest cross-sectional study of HRQoL in Swedish ESRD patients and so far the only study addressing HRQoL vs mortality among these patients.

We report that HRQoL indices correlated with some – but only a few - demographic, clinical and laboratory parameters that appeared to be linked to the well-being of the patients. Thus, following adjustments for age and sex, the physical component of HRQoL, PCS, associated positively with hemoglobin and serum albumin and negatively with age, CVD and DM, while the mental component of HRQoL, MCS, associated only with hemoglobin. Neither PCS nor MCS associated with eGFR. There were surprisingly few associations between MCS, and the numerous clinical factors assessed, suggesting we know little about the drivers of MCS and the items included in MCS. This should be an area for future studies.

Pagels et al. [10] in a study comprising 535 Swedish patients with CKD stages 2–5 reported that co-existing conditions, such as inflammation and cardiovascular disease seemed to be powerful predictors of impaired HRQoL. Other studies show that HRQoL is influenced by clinical and demographic factors such as age [25, 41] and gender [42], comorbidity, such as CVD [43] and DM [25], malnutrition [44], inflammation [44], anemia [45], and also residual renal function [46].

Poor HRQoL predicted all-cause mortality, but this association was confined to PCS in incident patients. In contrast, in a study on incident PD patients, not only low PCS but also low MCS associated with high mortality [18]. And, in a study in mainly diabetic patients [17], low MCS at dialysis initiation but neither low PCS scores nor Karnofsky scale associated with worse survival. In the NECOSAD Study [28], at 3 months after the start of dialysis, a mental SF-36 derived HRQoL score of 2-SD or less than the corresponding score in the general population predicted greater risk for poor outcome, but similar to the current study, a low MCS at baseline did not associate with mortality. These differences could be due to differences between investigated populations in terms of age, sex or comorbidities (like DM) but may also reflect different stages of the dialysis trajectory.

While HRQoL values as assessed by calculated effect sizes for differences were, as expected, lower in our ESRD patients than in a Swedish reference population [33, 36] they were lower also compared to previous results reported for prevalent dialysis patients [29, 47, 48]. It should be noted that most (84%) of our ESRD patients were incident dialysis patients. Due to the design, context and setting of the current study, analysis of differences between incident and prevalent dialysis patients was deemed not to be appropriate and, in any case, difficult to interpret considering differences among others in selection and clinical characteristics. Nevertheless, in the current study, the prevalent patients had higher MCS and, according to effect sizes (ES) of differences in HRQoL domains and summary scores, appeared to be better off than the incident dialysis patients. Similarly, lower PCS associated with increased all-cause mortality risk in the incident dialysis patients, whereas neither lower PCS nor lower MCS associated with all-cause mortality in the prevalent patients. These differences may reflect a more stable situation in the prevalent PD patients as compared to the incident dialysis patients.

Several studies examined HRQoL in PD patients [8], and some compared PD with HD [49–51]; however, a caveat for such comparisons is that differences between PD and HD may reflect different policies for starting patients on the respective treatment rather than differences between the therapies [52]. As mortality rate is higher in the period after start of hemodialysis [53], our prevalent PD patients constitute a selected group of clinically stable survivors, and this may also explain why HRQoL in the PD patients were better than HRQoL in the incident dialysis patients.

On the other hand, dialysis is a time-consuming procedure and as time passes, patients may become more burdened not only by complications linked to their kidney disease, but also by the time dealing with dialysis therapy and the way it interferes with the daily life, and by frustrations of feeling of being a burden on the family [20]. In a prospective cohort study [12], HRQoL declined steadily during a two-year study period; general health symptoms/problems, emotional well-being, burden of kidney disease, and patient satisfaction were the most significantly declined domains. In a multi-centre prospective cohort study, BRAZPD [18], PD patients with baseline HRQoL assessment had reduced HRQoL scores in some domains, demonstrating that HRQoL is already reduced at initiation of dialysis therapy. Furthermore, in the BRAZPD study, SF-36 scores of incident PD patients remained unchanged 12 months after the initiation of PD therapy. In our study, HRQoL was more impaired among the incident patients at the time of dialysis initiation than in those who had been treated 1 year on PD. Altogether these observations suggest that dialysis-initiating periods are critical transitional states during which patients may feel more vulnerable and anxious. This could be a reason explaining why the incident dialysis patients in our study had lower MCS than the prevalent PD patients.

We report that age was negatively associated with PCS (rho = − 0.17) in incident patients and (rho = − 0.45) in prevalent PD patient. This agrees with Griva et al. [41], who found that older patients showed significantly better HRQoL than younger patients despite their worse clinical profile. Levels of anxiety and depression were also lower in elderly patients. As shown by Harris et al. [54] and Griva et al. [41], elderly patients often report significantly better HRQoL than younger patients. Older and younger patients may have differential health expectations and ability to accept having to adapt to a worsening health status. Younger patients may have increased expectations of self-sufficiency and/or expectations to maintain or resume normal life style while older patients have lower illness/treatment intrusion, less social life to be disturbed, and better developed coping mechanisms, judging by the things that they already have accomplished.

Knowledge about the risk factors for poor HRQoL can guide identification of vulnerable ESRD patients and development of interventions to help them. Certain determinants of HRQoL in ESRD patients, not only clinical factors but also mental factors, are potentially modifiable [23, 55]. Early identification and correction of such factors may potentially lead to improving the overall well-being of patients. The results of the current study suggest that patients close to dialysis initiation are especially vulnerable because this phase of the disease trajectory presents many uncertainties to the patient [10] and therefore corrective, educational and supportive interventions are warranted during this period*.* While our study demonstrates differences such as between incident dialysis patients and prevalent PD patients, physical vs mental domains, and younger vs older patients, other factors influencing HRQoL, such as good self-recognition, may not be adequately captured by the SF-36 survey.

When interpreting these results some limitations of our study should be considered. First, this was an exploratory hypothesis generating analysis and because of the observational design of the study, no conclusions can be made regarding causality. Second, because of selection biases for inclusion of incident dialysis patients starting on HD (≈68%) and PD (≈32%) and prevalent PD patients, we cannot draw any conclusions regarding possible differences between these two groups, and the findings cannot necessarily be extrapolated to other dialysis patient populations. Third, this was an exploratory post hoc analysis of baseline data, and the evolution of HRQoL over time was not followed; this would be needed for interpreting illness trajectories. Fourth, we did not investigate a range of other potential determinants of HRQoL such as direct measurements of residual renal function, depression, fatigue, self-efficacy, coping strategies, socioeconomic aspects, employment, education level, loss of sexual function and marital discord, factors that might have influenced HRQoL directly, or indirectly. Fifth, the Swedish normative population used as a reference was not designed for the purpose of the present study. Sixth, because some of the individual components forming PCS are negatively weighted in calculating the MCS, and vice versa, this makes the interpretation of results more difficult. Finally, comparisons between the two cohorts should be interpreted with caution because of differences in sample size between incident (*n* = 338) and prevalent (*n* = 62) patients which together with differences in selection criteria and baseline characteristics, such as PD patients being in general more independent and having higher functional capacity than those who choose HD, precludes stringent statistical comparisons. Among strengths, it should be noted that patients were followed for up to 5 years and no patient was lost for follow-up.

## Conclusions

HRQoL as might be expected was lower in ESRD patients than in a normative Swedish population, and – also as expected – appeared to be lower in incident as compared to more clinically stable prevalent dialysis patients, suggesting that HRQoL may improve following adaption to dialysis treatment. However, no conclusions can be made regarding HRQoL in incident as compared to prevalent dialysis patients, due to the many differences between the two cohorts. Lower HRQoL associated with presence of comorbidities but also with a few factors such as lower hemoglobin and serum albumin concentrations, suggesting perhaps that these potentially modifiable factors could be relevant for HRQoL. MCS was not clearly associated to any of the investigated clinical factors, indicating that our knowledge about factors driving MCS is very limited. Lower HRQoL (PCS, but not MCS) associated with increased all-cause 5 years mortality in the incident patients, indicating that HRQoL - in addition to its obvious central role as a key measure of patient-centered outcomes – is of importance also for hard clinical outcomes in ESRD patients. Larger clinical studies are needed to assess factors determining HRQoL, and its association with clinical outcomes, in incident and prevalent dialysis patients, and to translate results into measures in clinical care aiming at improving the quality of care for dialysis patients.

## Additional file

Additional file 1: **Table S1.** Principal component analysis to determine construct validity for the two composite summary scales (PCS and MCS) of the SF-36 assessment in two cohorts. (DOCX 14 kb)

## Abbreviations

BP: Bodily pain
BRAZPD: Brazilian prospective peritoneal dialysis study
CKD: Chronic kidney disease
CKD-EPI: CKD Epidemiology Collaboration formula for estimated glomerular filtration rate
CRP: C-reactive protein
CVD: Cardiovascular disease
DM: Diabetes mellitus
eGFR: estimated glomerular filtration rate
ES: standardized measure of effect size
ESRD: End-stage renal disease
GH: General health
HD: Hemodialysis
HRQoL: Health-related quality of life
IL-6: Interleukin-6
iPTH: intact parathyroid hormone
MCS: Health-related quality of life mental composite score
MH: Mental health/emotional well-being role limitations caused by mental health/emotional problems
MIMICK2: Mapping of Inflammation Markers in Chronic Kidney Disease 2
PAUS: Prospective study of renal replacement therapy in Stockholm
PCS: Health-related quality of life physical composite score
PD: Peritoneal dialysis
PF: Physical functioning
RP: Role limitations caused by physical problems
SF: Social functioning
SF-36: Short Form-36
VT: Vitality/energy/fatigue

## References

[1] Stenvinkel P, Larsson TE (2013). Chronic kidney disease: a clinical model of premature aging. Am J Kidney Dis.

[2] Kooman JP, Kotanko P, Schols AM, Shiels PG, Stenvinkel P (2014). Chronic kidney disease and premature ageing. Nat Rev Nephrol.

[3] Eckardt KU, Coresh J, Devuyst O, Johnson RJ, Kottgen A, Levey AS, Levin A (2013). Evolving importance of kidney disease: from subspecialty to global health burden. Lancet.

[4] Glover C, Banks P, Carson A, Martin CR, Duffy T (2011). Understanding and assessing the impact of end-stage renal disease on quality of life: a systematic review of the content validity of self-administered instruments used to assess health-related quality of life in end-stage renal disease. Patient.

[5] Tang E, Bansal A, Novak M, Mucsi I (2017). Patient-reported outcomes in patients with chronic kidney disease and kidney transplant-part 1. Front Med (Lausanne).

[6] Mapes DL, Lopes AA, Satayathum S, McCullough KP, Goodkin DA, Locatelli F, Fukuhara S, Young EW, Kurokawa K, Saito A (2003). Health-related quality of life as a predictor of mortality and hospitalization: the Dialysis Outcomes and Practice Patterns Study (DOPPS). Kidney Int.

[7] dos Santos Grincenkov FR, Fernandes N, Chaoubah A, da Silva Fernandes N, Bastos K, Lopes AA, Qureshi AR, Finkelstein FO, Pecoits-Filho R, Divino-Filho JC (2013). Longitudinal changes in health-related quality of life scores in Brazilian incident peritoneal dialysis patients (BRAZPD): socio-economic status not a barrier. Perit Dial Int.

[8] Aguiar R, Pei M, Qureshi AR, Lindholm B. Health-related quality of life in peritoneal dialysis patients: a narrative review. Semin Dial. 2018. 10.1111/sdi.12770 [Epub ahead of print]. 10.1111/sdi.12770 30575128

[9] Mujais SK, Story K, Brouillette J, Takano T, Soroka S, Franek C, Mendelssohn D, Finkelstein FO (2009). Health-related quality of life in CKD patients: correlates and evolution over time. Clin J Am Soc Nephrol.

[10] Pagels AA, Soderkvist BK, Medin C, Hylander B, Heiwe S (2012). Health-related quality of life in different stages of chronic kidney disease and at initiation of dialysis treatment. Health Qual Life Outcomes.

[11] Sorensen VR, Mathiesen ER, Watt T, Bjorner JB, Andersen MV, Feldt-Rasmussen B (2007). Diabetic patients treated with dialysis: complications and quality of life. Diabetologia.

[12] Bakewell AB, Higgins RM, Edmunds ME (2002). Quality of life in peritoneal dialysis patients: decline over time and association with clinical outcomes. Kidney Int.

[13] Soni RK, Weisbord SD, Unruh ML (2010). Health-related quality of life outcomes in chronic kidney disease. Curr Opin Nephrol Hypertens.

[14] Shimoyama S, Hirakawa O, Yahiro K, Mizumachi T, Schreiner A, Kakuma T (2003). Health-related quality of life and caregiver burden among peritoneal dialysis patients and their family caregivers in Japan. Perit Dial Int.

[15] Griva K, Goh CS, Kang WCA, Yu ZL, Chan MC, Wu SY, Krishnasamy T, Foo M (2016). Quality of life and emotional distress in patients and burden in caregivers: a comparison between assisted peritoneal dialysis and self-care peritoneal dialysis. Qual Life Res.

[16] Thong MS, Kaptein AA, Benyamini Y, Krediet RT, Boeschoten EW, Dekker FW, Netherlands Cooperative Study on the Adequacy of Dialysis Study G (2008). Association between a self-rated health question and mortality in young and old dialysis patients: a cohort study. Am J Kidney Dis.

[17] Lopez Revuelta K, Garcia Lopez FJ, de Alvaro Moreno F, Alonso J (2004). Perceived mental health at the start of dialysis as a predictor of morbidity and mortality in patients with end-stage renal disease (CALVIDIA study). Nephrol Dial Transplant.

[18] Grincenkov FR, Fernandes N, Pereira Bdos S, Bastos K, Lopes AA, Finkelstein FO, Pecoits-Filho R, Qureshi AR, Divino-Filho JC, Bastos MG (2015). Impact of baseline health-related quality of life scores on survival of incident patients on peritoneal dialysis: a cohort study. Nephron.

[19] Bossola M, Pepe G, Marzetti E (2017). Health-related quality of life of patients on chronic dialysis: the need for a focused effort. Semin Dial.

[20] Guo Amy, Wolfson Marsha, Holt Robert (2002). Early quality of life benefits of icodextrin in peritoneal dialysis. Kidney International.

[21] Iyasere O, Brown EA (2014). Determinants of quality of life in advanced kidney disease: time to screen?. Postgrad Med J.

[22] Bakewell AB, Higgins RM, Edmunds ME (2001). Does ethnicity influence perceived quality of life of patients on dialysis and following renal transplant?. Nephrol Dial Transplant.

[23] Kim JY, Kim B, Park KS, Choi JY, Seo JJ, Park SH, Kim CD, Kim YL (2013). Health-related quality of life with KDQOL-36 and its association with self-efficacy and treatment satisfaction in Korean dialysis patients. Qual Life Res.

[24] Yang F, Griva K, Lau T, Vathsala A, Lee E, Ng HJ, Mooppil N, Foo M, Newman SP, Chia KS (2015). Health-related quality of life of Asian patients with end-stage renal disease (ESRD) in Singapore. Qual Life Res.

[25] Grincenkov FR, Fernandes N, Chaoubah A, Bastos K, Qureshi AR, Pecoits-Filho R, Divino Filho JC, Bastos MG (2011). Factors associated with the quality of life of incident patients on PD in Brazil (BRAZPD). J Bras Nefrol.

[26] Wu AW (2004). Changes in quality of life during hemodialysis and peritoneal dialysis treatment: generic and disease specific measures. J Am Soc Nephrol.

[27] Senol V, Sipahioglu MH, Ozturk A, Argun M, Utas C (2010). Important determinants of quality of life in a peritoneal dialysis population in Turkey. Ren Fail.

[28] Merkus MP, Jager KJ, Dekker FW, de Haan RJ, Boeschoten EW, Krediet RT (2000). Predictors of poor outcome in chronic dialysis patients: the Netherlands Cooperative Study on the Adequacy of Dialysis. The NECOSAD Study Group. Am J Kidney Dis.

[29] Merkus MP, Jager KJ, Dekker FW, De Haan RJ, Boeschoten EW, Krediet RT (1999). Quality of life over time in dialysis: the Netherlands Cooperative Study on the Adequacy of Dialysis. NECOSAD Study Group. Kidney Int.

[30] Okaka EI, Davies M, Ahmed M, Naidoo S, Naicker S (2014). Impact of socio-economic factors on quality of life in patients on continuous ambulatory peritoneal dialysis in an African setting. West Afr J Med.

[31] Boateng EA, East L (2011). The impact of dialysis modality on quality of life: a systematic review. J Ren Care.

[32] Effect Size Calculators. http://www.uccs.edu/~lbecker/. Accessed 14 Apr 2016.

[33] Sullivan M, Karlsson J, Taft C (2002). Hälsoenkät: Svensk Manual och Tolkningsguide.

[34] Xu H, Cabezas-Rodriguez I, Qureshi AR, Heimburger O, Barany P, Snaedal S, Anderstam B, Helin AC, Carrero JJ, Stenvinkel P (2015). Increased levels of modified advanced oxidation protein products are associated with central and peripheral blood pressure in peritoneal dialysis patients. Perit Dial Int.

[35] Ware JE, Sherbourne CD (1992). The MOS 36-item short-form health survey (SF-36). I. Conceptual framework and item selection. Med Care.

[36] Sullivan M, Karlsson J (1998). The Swedish SF-36 Health Survey III. Evaluation of criterion-based validity: results from normative population. J Clin Epidemiol.

[37] Magder SA (2014). The highs and lows of blood pressure: toward meaningful clinical targets in patients with shock. Crit Care Med.

[38] Davies SJ, Phillips L, Naish PF, Russell GI (2002). Quantifying comorbidity in peritoneal dialysis patients and its relationship to other predictors of survival. Nephrol Dial Transplant.

[39] Levey AS, Stevens LA, Schmid CH, Zhang YL, Castro AF, Feldman HI, Kusek JW, Eggers P, Van Lente F, Greene T (2009). A new equation to estimate glomerular filtration rate. Ann Intern Med.

[40] Filiopoulos V, Koutis I, Takouli L, Arvanitis D, Panagiotopoulos K, Vlassopoulos D (2013). Chronic kidney disease epidemiology collaboration equation accuracy in predicting peritoneal dialysis-delivered creatinine clearance. Ren Fail.

[41] Griva K, Yu Z, Chan S, Krisnasamy T, Yamin RB, Zakaria FB, Wu SY, Oei E, Foo M (2014). Age is not a contraindication to home-based dialysis - quality-of-life outcomes favour older patients on peritoneal dialysis regimes relative to younger patients. J Adv Nurs.

[42] Mingardi G, Cornalba L, Cortinovis E, Ruggiata R, Mosconi P, Apolone G (1999). Health-related quality of life in dialysis patients. A report from an Italian study using the SF-36 Health Survey. DIA-QOL Group. Nephrol Dial Transplant.

[43] Okubo R, Kai H, Kondo M, Saito C, Yoh K, Morito N, Usui J, Yamagata K (2014). Health-related quality of life and prognosis in patients with chronic kidney disease: a 3-year follow-up study. Clin Exp Nephrol.

[44] Bilgic A, Akman B, Sezer S, Ozisik L, Arat Z, Ozdemir FN, Haberal M (2008). Predictors for quality of life in continuous ambulatory peritoneal dialysis patients. Nephrology (Carlton).

[45] Finkelstein FO, Story K, Firanek C, Mendelssohn D, Barre P, Takano T, Soroka S, Mujais S (2009). Health-related quality of life and hemoglobin levels in chronic kidney disease patients. Clin J Am Soc Nephrol.

[46] Termorshuizen F, Korevaar JC, Dekker FW, van Manen JG, Boeschoten EW, Krediet RT, Group NS (2003). The relative importance of residual renal function compared with peritoneal clearance for patient survival and quality of life: an analysis of the Netherlands Cooperative Study on the Adequacy of Dialysis (NECOSAD )-2. Am J Kidney Dis.

[47] Korevaar JC, Jansen MA, Merkus MP, Dekker FW, Boeschoten EW, Krediet RT (2000). Quality of life in predialysis end-stage renal disease patients at the initiation of dialysis therapy. The NECOSAD Study Group. Perit Dial Int.

[48] Lee AJ, Morgan CL, Conway P, Currie CJ (2005). Characterisation and comparison of health-related quality of life for patients with renal failure. Curr Med Res Opin.

[49] Fong E, Bargman JM, Chan CT (2007). Cross-sectional comparison of quality of life and illness intrusiveness in patients who are treated with nocturnal home hemodialysis versus peritoneal dialysis. Clin J Am Soc Nephrol.

[50] Brown EA, Johansson L, Farrington K, Gallagher H, Sensky T, Gordon F, Da Silva-Gane M, Beckett N, Hickson M (2010). Broadening Options for Long-term Dialysis in the Elderly (BOLDE): differences in quality of life on peritoneal dialysis compared to haemodialysis for older patients. Nephrol Dial Transplant.

[51] Makkar V, Kumar M, Mahajan R, Khaira NS (2015). Comparison of outcomes and quality of life between hemodialysis and peritoneal Dialysis patients in Indian ESRD population. J Clin Diagn Res.

[52] Ghaffari A, Kalantar-Zadeh K, Lee J, Maddux F, Moran J, Nissenson A (2013). PD first: peritoneal dialysis as the default transition to dialysis therapy. Semin Dial.

[53] Eckardt KU, Gillespie IA, Kronenberg F, Richards S, Stenvinkel P, Anker SD, Wheeler DC, de Francisco AL, Marcelli D, Froissart M (2015). High cardiovascular event rates occur within the first weeks of starting hemodialysis. Kidney Int.

[54] Harris SA, Lamping DL, Brown EA, Constantinovici N, North Thames Dialysis Study G (2002). Clinical outcomes and quality of life in elderly patients on peritoneal dialysis versus hemodialysis. Perit Dial Int.

[55] Jhamb M, Weiner DE (2014). Exercise to improve physical function and quality of life in CKD. Clin J Am Soc Nephrol.

