# Supplementary material for: Health-related quality of life as predictor of mortality in end-stage renal disease patients: an observational study
Source: BMC Nephrol. 2019 Apr 29;20:144. doi: 10.1186/s12882-019-1318-x (PMC6489294; doi:10.1186/s12882-019-1318-x)
Supplement: Supplementary file 1 — Table S1. Principal component analysis to determine construct validity for the two composite summary scales (PCS and MCS) of the SF-36 assessment in two cohorts. (DOCX 14 kb) [file 12882_2019_1318_MOESM1_ESM.docx]

**Additional file 1 Table S1.** Principal component analysis to determine construct validity for the two composite summary scales (PCS and MCS) of the SF-36 assessment in two cohorts.

|  | Rotated principal components | | Rotated principal components | |
| --- | --- | --- | --- | --- |
|  | Incident patients, n=338 | | Prevalent PD patients, n=62 | |
|  | Physical† | Mental† | Physical† | Mental† |
| Physical Functioning (PF) | -0.07 | -0.26 | -0.05 | -0.08 |
| Role Physical (RP) | 0.30 | 0.17 | 0.10 | 0.07 |
| Bodily Pain (BP) | 0.17 | 0.02 | 0.11 | 0.06 |
| General Health (GH) | 0.19 | 0.01 | 0.42 | 0.33 |
| Vitality (VT) | 0.42 | 0.29 | 0.41 | 0.33 |
| Social Functioning (SF) | 0.33 | 0.22 | 0.44 | 0.38 |
| Role Emotional (RE) | 0.50 | 0.44 | 0.29 | 0.28 |
| Mental Health (MH) | 0.53 | 0.46 | 0.59 | 0.50 |
| Eigenvalue | 3.71 | 3.13 | 3.16 | 3.91 |
| Variance % | 75.2 | 74.2 | 35.2 | 43.4 |

Abbreviations: †Correlation between each SF-36 scale and rotated principal component. Strong association. r ≥ 0.70; moderate to substantial association. 0.30 < r > 0.70; weak association. r ≤ 0.30.

Interpretation: In the SF-36 model, the PF, BP, and RP scales loaded on the physical component as expected, while the GH scale loaded on the mental instead of the physical component. Also as expected, the MH. RE and VT scales loaded on the mental component. while the SF scale loaded on the physical component instead of the mental component.
